# Supplementary material for: Genome-Wide Characterization of bHLH Family Genes and Expression Analysis in Response to Osmotic Stress in Betula platyphylla
Source: Plants (Basel). 2023 Oct 25;12(21):3687. doi: 10.3390/plants12213687 (PMC10649471; doi:10.3390/plants12213687)

Table S1. Prediction of the size, isoelectric point and subcellular localization of bHLH family proteins

Protien ID	Number of amino acid	Molecular weight	Isoelectric point, pI	Grand average of hydropathicity	Subcellular localization
BPChr01G13692	230	25661.28	5.45	-0.388	nucleus
BPChr01G18056	432	46593.74	5.88	-0.626	nucleus
BPChr01G20319	221	23734.29	5.39	-0.635	nucleus
BPChr01G22813	309	35215.05	4.67	-0.432	nucleus
BPChr01G22934	367	40677.77	8.97	-0.544	nucleus
BPChr01G25595	344	38366.63	5.07	-0.647	nucleus
BPChr02G00309	494	53253.25	6.19	-0.621	nucleus
BPChr02G13051	247	27978.77	5.59	-0.416	nucleus
BPChr02G13119	248	28110.85	5.6	-0.463	nucleus
BPChr02G13124	258	29294.85	6	-0.551	nucleus
BPChr02G19943	258	27678.94	7.62	-0.55	nucleus
BPChr02G19959	896	98136.15	5.04	-0.282	nucleus
BPChr02G20693	669	72828.93	5.35	-0.584	nucleus
BPChr02G20825	321	35823.85	7.1	-0.334	nucleus
BPChr02G23304	542	59129.14	5.35	-0.549	nucleus
BPChr02G23454	346	38386.67	9.3	-0.608	nucleus
BPChr02G23542	239	27436.39	8.52	-0.579	nucleus
BPChr02G25935	712	79889.6	5.54	-0.365	nucleus
BPChr03G00992	745	82426.11	5.84	-0.359	nucleus
BPChr03G03233	288	31789.17	5.3	-0.634	nucleus
BPChr03G13906	214	24217.01	5.65	-0.712	nucleus
BPChr03G15668	530	60388.12	6.92	-0.488	nucleus
BPChr03G19797	506	55874.69	5.75	-0.442	nucleus
BPChr04G00593	249	27763.23	5.64	-0.762	nucleus
BPChr04G03663	91	10240.58	7.94	-0.537	nucleus
BPChr04G04974	93	10491.8	7.9	-0.655	nucleus
BPChr04G09407	454	49703.13	9.25	-0.639	nucleus
BPChr04G23830	393	42933.02	7.11	-0.501	nucleus
BPChr04G24004	208	23196.05	9.49	-0.101	chloroplast
BPChr04G25971	360	38887.4	5.46	-0.577	nucleus
BPChr04G27162	236	25297.45	8.69	-0.469	nucleus
BPChr04G31973	229	25474.67	5.82	-0.769	nucleus
BPChr05G04602	348	38447.97	6.06	-0.887	nucleus
BPChr05G08772	323	35888.61	5.08	-0.303	nucleus
BPChr05G08785	722	80545.95	5.57	-0.672	nucleus
BPChr05G14657	437	48283.73	6.16	-0.434	nucleus
BPChr05G21072	164	17876.53	8.88	-0.269	nucleus
BPChr05G21100	208	22808.48	11.3	-0.688	nucleus

BPChr05G22447	347	38867.74	6.21	-0.508	nucleus
BPChr05G22456	198	21881.25	11.51	-0.651	nucleus
BPChr05G22500	349	38503.5	7.63	-0.497	nucleus
BPChr05G27408	463	50940.34	8.8	-0.672	nucleus
BPChr05G31810	331	35346.98	5.84	-0.324	nucleus
BPChr06G02661	422	46741.46	8.18	-0.554	nucleus
BPChr06G08058	299	32919.74	6.23	-0.596	nucleus
BPChr06G09444	254	28404.08	7.07	-0.533	nucleus
BPChr06G09455	244	27420.76	5.81	-0.419	nucleus
BPChr06G09470	281	31550.66	8.87	-0.66	nucleus
BPChr06G09475	237	25720.16	7.64	-0.538	nucleus
BPChr06G09578	459	49967.28	6.19	-0.614	nucleus
BPChr06G16498	440	48323.18	5.38	-0.387	nucleus
BPChr06G16504	349	39149.28	9.49	-0.436	nucleus
BPChr06G21431	499	55687.51	5.36	-0.741	nucleus
BPChr06G21469	475	52980.57	5.54	-0.721	nucleus
BPChr06G29381	428	48181.93	5.3	-0.637	nucleus
BPChr06G29401	667	74948.6	5.01	-0.437	nucleus
BPChr06G29449	483	54407.48	5.78	-0.526	nucleus
BPChr06G29488	611	68173.95	7.2	-0.538	nucleus
BPChr06G29579	379	42896.69	6.1	-0.514	nucleus
BPChr06G30385	260	28626.52	7.58	-0.398	nucleus
BPChr06G30704	337	37634.89	5.18	-0.557	nucleus
BPChr06G30727	337	37634.89	5.18	-0.557	nucleus
BPChr06G31065	260	28626.52	7.58	-0.398	nucleus
BPChr07G10012	280	30472.23	6.41	-0.465	nucleus
BPChr07G15610	208	23553.99	5.91	-0.419	nucleus
BPChr07G15621	476	52047.53	5.78	-0.593	nucleus
BPChr07G15655	216	23932.33	5.36	-0.469	nucleus
BPChr07G17347	243	27145.04	5.04	-0.728	nucleus
BPChr07G18813	181	20528.96	6.32	-0.738	nucleus
BPChr08G01250	303	32413.77	5.53	-0.74	nucleus
BPChr08G01254	94	10588.85	9.09	-0.63	nucleus
BPChr08G04985	194	21751.33	8.17	0.001	chloroplast, mitochondrion
BPChr08G11237	168	19167.1	8.3	-1.198	nucleus
BPChr08G11303	551	59163.56	5.3	-0.627	nucleus
BPChr08G11341	436	48147.3	6.11	-0.611	nucleus
BPChr08G15294	526	56807.91	6.15	-0.68	nucleus
BPChr08G16111	225	25008.89	9.76	-0.437	chloroplast
BPChr08G16126	282	31164.47	7.06	-0.352	nucleus
BPChr08G16198	742	79482.58	5.87	-0.489	nucleus

BPChr08G19072	282	30968.34	6.2	-0.624	nucleus
BPChr08G22987	492	55224.7	5.92	-0.458	nucleus
BPChr08G24211	339	37514.27	5.05	-0.428	nucleus
BPChr08G24314	439	47768.14	7.74	-0.548	nucleus
BPChr09G01140	357	39012.84	4.95	-0.763	nucleus
BPChr09G01155	307	34084.17	4.85	-0.534	nucleus
BPChr09G01161	331	36478.58	4.85	-0.585	nucleus
BPChr09G01651	492	54809.7	8.81	-0.086	endomembrane, plasma membrane
BPChr09G06515	216	23878.32	5.32	-0.448	nucleus
BPChr09G12090	230	26673.47	8.9	-0.689	nucleus
BPChr09G16382	633	71473.12	5.7	-0.552	extracellular space, nucleus
BPChr09G20024	299	32351.23	7.72	-0.518	nucleus
BPChr09G20526	452	50882.71	6.88	-0.454	chloroplast
BPChr09G20534	440	49517.35	7.19	-0.444	nucleus
BPChr09G20958	277	30298.21	6.84	-0.672	nucleus
BPChr09G29950	348	38545.19	5.51	-0.608	nucleus
BPChr09G29953	411	45207.55	5.37	-0.541	nucleus
BPChr10G03484	255	27992.93	6.54	-0.232	nucleus
BPChr10G21682	938	102435.4	6.4	-0.405	nucleus
BPChr10G28285	401	43455.9	5.62	-0.724	nucleus
BPChr10G28366	438	47504.81	6.09	-0.624	nucleus
BPChr11G02096	161	18312.16	7.6	-0.034	nucleus
BPChr11G04400	303	33771.12	6.75	-0.376	nucleus
BPChr11G05889	66	7346.39	5.65	-0.28	nucleus
BPChr11G07048	233	26440.73	5.32	-0.555	nucleus
BPChr11G07162	238	27099.7	5.2	-0.472	nucleus
BPChr11G10166	217	24680.08	5.89	-0.483	nucleus
BPChr11G12210	546	59216.54	5.15	-0.442	nucleus
BPChr11G17797	487	53706.02	5.91	-0.679	nucleus
BPChr12G11347	91	10240.58	7.94	-0.537	nucleus
BPChr12G11533	331	36442	5.8	-0.424	nucleus
BPChr12G13409	616	67680.79	8.72	-0.667	nucleus
BPChr12G22339	471	49713.21	6.21	-0.604	nucleus
BPChr12G24135	309	34735.36	6.68	-0.515	nucleus
BPChr12G25787	239	26511.36	9.1	-0.506	nucleus
BPChr12G25898	362	39397.57	5.01	-0.588	nucleus
BPChr13G07710	388	43085.27	8.13	-0.697	nucleus
BPChr13G10317	499	53933.74	5.99	-0.64	nucleus
BPChr13G16040	558	60924.19	8.36	-0.478	nucleus

BPChr13G16604	319	36039.02	9.41	-0.5	nucleus
BPChr14G06667	1487	164407.6	6.07	-0.333	chloroplast
BPChr14G12524	242	27146.83	6.19	-0.442	nucleus
BPChr14G12967	322	35299.65	7.12	-0.642	nucleus
BPChr14G24216	158	17545.73	8.53	-0.539	nucleus
BPChr14G27000	247	27978.77	5.59	-0.416	nucleus
BPChr14G27081	258	29294.85	6	-0.551	nucleus
BPChr14G27093	248	28110.85	5.6	-0.463	nucleus
BPunChr33545	331	37461.51	5.29	-0.308	nucleus

Table S2 Primers for eight BpbHLH genes

Protien ID	Name	Sequence
BPChr11G07048	*BpbHLH104*	F:GTATGGACAGTTGGCAATTC
		R:GATATCGAAATGATAAACCAGT
BPChr06G09470	*BpbHLH060*	F:CAGGTTCTCAATCTGGTCC
		R:CCACTCGTTCGATGCCGT
BPChr06G09475	*BPbHLH056*	F:ACAGCACACTTCAGATCTAC
		R:TCTACGATTACGGCGTGATC
BPChr08G11237	*BPbHLH080*	F:ATGAGAACAGGAAAGGGTAAT
		R:TACCTCTCGTTGATTTTGCT
BPChr08G16126	*BpbHLH076*	F:GAATCGTCTTCAACCTTAACA
		R:CATTCAGCCATTGCAGACAT
BPChr01G18056	*BpbHLH004*	F:GCTTCCATGCTTTGGAAGTG
		R:GCGAAGATCCTATAGCACCCT

BPChr12G24135	*BpbHLH115*	F:TACTTTCATGGAATTCATTTCC
		R:GACAACTTCCGACATGGATT
BPChr12G25787	*BpbHLH111*	F:GCTATGCGGAAGATCATCTC
		R:GGCAACAAAATGGGTGCCAT

Figure S1 Genes Phylogenetic analysis in Response to osmosis Stress

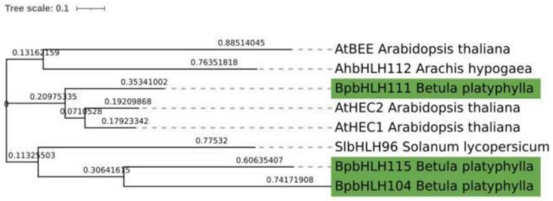

Figure S2 Phylogenetic analysis

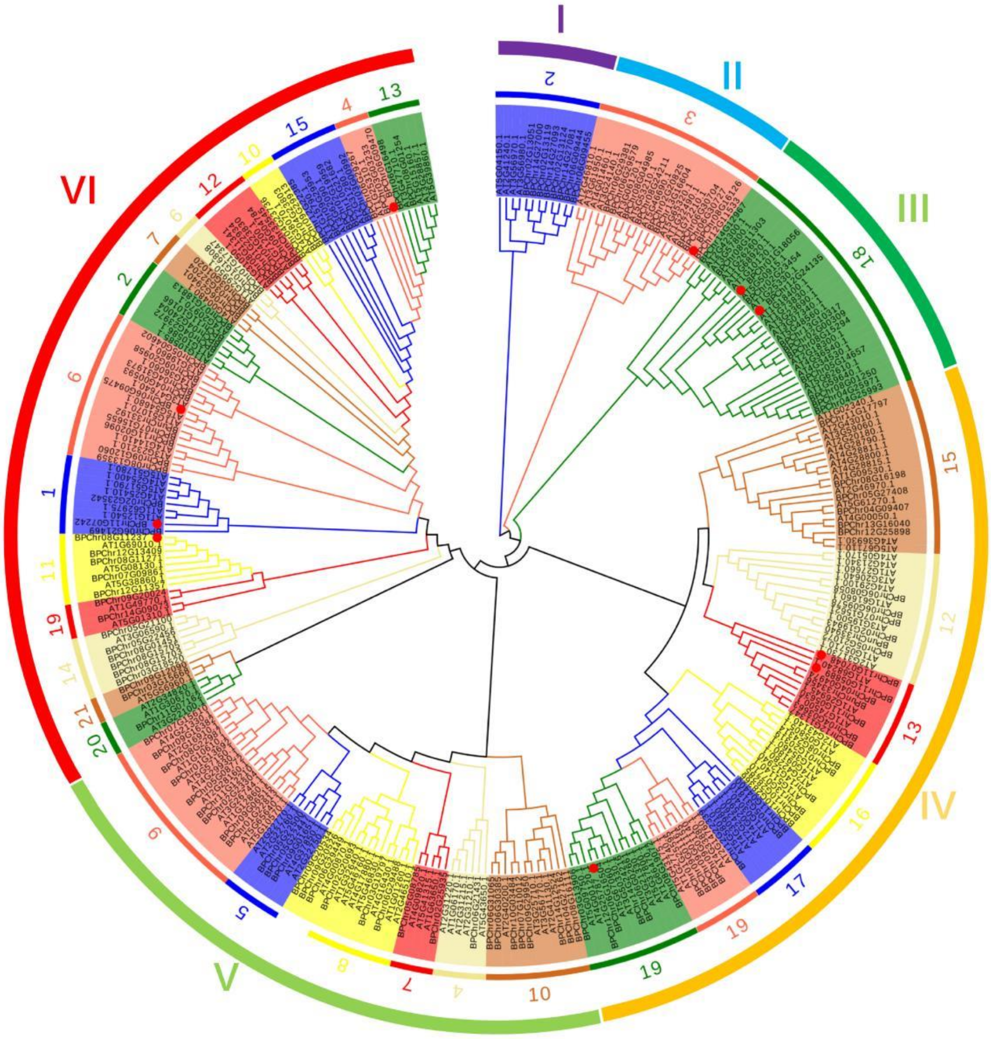

Supplement: Supplementary file 1 [file plants-12-03687-s001.zip › plants-2576463-supplementary.pdf]
